# Impact of Arm Dominance and Location on Detecting Electrotactile Stimuli during Voluntary Motor Activation in Older Adults

**DOI:** 10.1109/TOH.2023.3268203

**Published:** 2023-12-21

**Authors:** Ninghe M. Cai, Eileen G. Medina, Stefani Gonzalez, Alan Duong, Netta Gurari

**Affiliations:** 1 NMC, SG, AG, and NG are with the Department of Physical Therapy and Human Movement Sciences, Northwestern University, 645 N Michigan Ave Suite 1100, Chicago, IL, 60611, USA; 2 EGM and NG are with the Department of Biomedical Engineering & Mechanics, Virginia Polytechnic Institute and State University, 495 Old Turner St, Blacksburg, VA, 24061, USA

## Abstract

Activation-related sensory gating is important for sensorimotor control, filtering signals irrelevant to a task. Literature on brain lateralization suggests that motor activation patterns during sensorimotor control differ depending on arm dominance. Whether the lateralization effect extends to how sensory signals modulate during voluntary sensorimotor control remains unaddressed. We compared tactile sensory gating during voluntary motor activation between the arms of older adults.

Eight right-arm dominant participants received a single-pulse, 100*μ*s square-wave electrotactile stimulus at their testing arm’s fingertip or elbow. We identified at both arms the threshold at which the electrotactile stimulus was detected when participants were at rest (baseline) and isometrically flexing about the elbow to 25% and 50% of their maximum voluntary torque.

Results reveal a difference in the detection threshold at the fingertip (p<0.001) between the arms, yet not the elbow (p=0.264). Additionally, results demonstrate that greater isometric flexion about the elbow yields increased detection thresholds at the elbow (p=0.005), yet not the fingertip (p=0.069). However, the changes in detection threshold during motor activation did not significantly differ between the arms (p=0.154).

The findings regarding an impact of arm dominance and location on tactile perception are important when considering sensorimotor perception and training, including post-unilateral injury.

## I. INTRODUCTION

Accurately perceiving tactile stimuli is necessary to seamlessly interact with and manipulate objects. Mundane tasks such as retrieving an object from a shelf are difficult, if not impossible, without accurate tactile perception. During movement, the human body is bombarded by somatosensory signals. As a result, mechanisms are in place to gate somatosensory signals so that the human can focus on the signals relevant to the task. Regardless of the origin of the somatosensory signal, whether it is self generated or externally applied, the sensory response is suppressed during voluntary motor activation [1], [2], [3], [4]. In other words, motor activation modulates the transmission and processing of sensory signals. Previous research has implicated a contribution of brain lateralization on differing aspects of sensorimotor control [5], [6]. As many activities of daily living, such as eating with a fork and knife, require the usage and coordination of both arms, it is important to understand whether there is a corresponding role of arm dominance, a consequence of brain lateralization, on the modulation of sensory signal transmission. In this work, we explore the role of brain lateralization on somatosensory signal transmission during voluntary motor activation by determining how externally-applied electrotactile stimuli are perceived.

When external tactile stimuli are applied during motor activation, sensory-evoked potentials are inhibited at subcortical and cortical sites along the somatosensory pathway [7], [8]. This mechanism has been observed across species, including in monkeys [9], [10] and mice [11]. The suppression of externally-generated tactile stimuli is observed in humans physiologically [12], [13], as well as behaviorally through an increase in a detection threshold [14], [15], [16]. Several mechanisms have been proposed to explain the increased detection threshold during motor activation [17]. One proposed mechanism suggests that somatosensory signals arising from the skin, joints, and muscles of the activated limb could mask external tactile stimuli. In turn, a tactile stimulus applied closer to the motor activation site may be suppressed to a greater degree [18]. Another proposed mechanism relates to sensorimotor prediction in that tactile signals congruent to a prediction are attenuated more strongly during movement [19]. Additional proposed mechanisms exist such as active inference [20] and the opposing process theory [21]. While the mechanism underlying tactile gating is debated, the role of tactile gating on sensorimotor control remains important, facilitating the execution of sensorimotor tasks.

Literature has implicated an effect of brain lateralization on sensorimotor control [22]. Specifically, the left hemisphere in right-arm dominant individuals is thought to specialize in predictable conditions, and the right hemisphere is thought to specialize in stabilizing during unpredictable events [5], [22]. With activation-related sensory gating playing an important role in sensorimotor control, brain lateralization could impact the suppression of tactile information [15]. Previous studies demonstrated that arm dominance affects spatial [23] and temporal [24] tactile perception. However, it is unclear whether arm dominance influences perception of tactile stimuli during motor activation. As a result, our study aims to address whether the impact of voluntary motor activation on tactile sensory gating is influenced by brain lateralization.

Of particular interest for our group’s ongoing research, we investigate the phenomenon of tactile gating in older adults. Populations with neurological impairments, such as stroke, who experience abnormal sensorimotor control, are often older in age. Therefore, our understanding of tactile gating relevant to these populations with neurological injuries could benefit from knowing expected behaviors in a similarly-aged cohort of older adults who are neurotypical.

We evaluated the threshold at which older adults could detect an externally-applied electrotactile stimulus at the fingertip and elbow of each arm during isometric elbow flexion. We hypothesized that the detection threshold at the fingertip and elbow in the non-dominant arm would increase to a greater extent than the dominant arm. This hypothesis arises from the brain lateralization model, particularly the notion that the non-dominant arm is specialized for unpredictable events where stabilization is the goal, rather than dexterous, fine-tuned movements [15]. Additionally, we hypothesized that the impact of sensory gating would be more evident at the elbow than the fingertip given the proximity of the elbow to the site of motor activation (i.e., elbow flexion). Understanding differences in tactile perception between arms during motor activation could have important implications for bimanual task manipulation and sensorimotor training.

## II. METHODS

### A. Participants

The Northwestern University Institutional Review Board approved this study (STU00208205). Nine older adults who were right-hand dominant consented to participate; one was excluded due to a sensory neuropathy. The eight participants [6 males, 2 females; age (mean±SD): 62±4] included in the study did not report any neurological and musculoskeletal injuries that would result in impaired tactile signal transmission.

### B. Experimental Setup

The participant sat in a Biodex chair (System 3 ProTM; Shirley, USA) with their torso strapped to limit movement. Their testing forearm was casted and attached to a mechatronic device at set angles of 45° shoulder flexion, 85° shoulder abduction, and 90° elbow flexion. The cast ensured a rigid connection between the testing arm and a 6 degree-of-freedom load cell (JR3, Model: 45E15A 1000N; Woodland, USA) that measured the torques applied by the participant.

The participant obtained visual feedback from a 42-inch screen monitor (Panasonic TH-42PH9, Osaka, Japan) and audio feedback from speakers. The visual feedback provided real-time information about the torque generated by the participant, as well as information on the target torque. The red circle reflected the torque generated by the participant; its diameter increased and decreased corresponding to a respective increase and decrease in the torque magnitude generated. The black circle represented the target torque and the inner and outer blue circles identified the range of allowable elbow torques. The desired range was defined as the target elbow flexion torque±5% MVT (maximum voluntary torque).

**Fig. 1.**
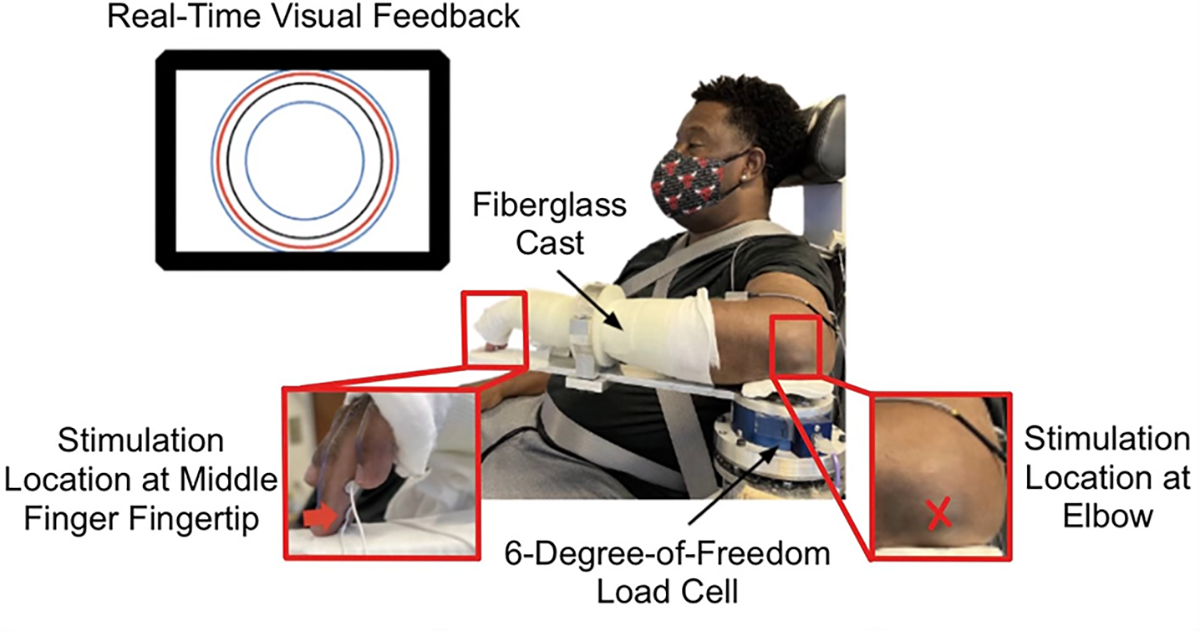
Experimental Setup. The participant’s testing arm was affixed to a mechatronic device. The gray adhesive element depicted in the red box on the left displays the location on the testing arm’s middle fingertip at which the electrotactile stimulus was provided. The red “X” depicted in the red box on the right displays the location on the testing arm’s elbow at which the electrotactile stimulus was provided. The participant received real-time visual feedback on their self-generated (red circle) elbow flexion torque, as measured by the load cell, as well as the target (black circle) and range of allowable (blue circles) elbow torques.

A constant current stimulator (DIGITIMER, Model: DS7A; Welwyn Garden City, England) delivered electrotactile stimuli to the participant. The stimulator electrode was placed at the following two locations of the arm tested: 1) palmar aspect of the middle finger’s fingertip and 2) protruded aspect of the elbow’s olecranon (Fig 1). For each location, the ground electrode was placed one inch proximal to the stimulator electrode. The middle finger was chosen for its ability to stimulate the median nerve. Stimulation at the elbow targeted the ulnar nerve. The electrotactile stimulus was delivered as a single 100 *μ*s square-wave pulse with a maximum amplitude of 100 mA to target only the somatosensory pathway, avoiding the pain and motor pathways [25].

The software updated at 4 kHz, and data summarizing the experimental protocol (e.g., time, torque, electrotactile stimulus) were saved at 1 kHz.

### C. Maximum Voluntary Torque

The participant’s MVT in elbow flexion was obtained. The participant maximally flexed about their elbow for five seconds, and the experimenter verbally encouraged the participant throughout. This task was repeated at least three times and stopped when the maximum elbow flexion torque values obtained were within 10% of each other and the last trial was not maximal. Real-time visual feedback depicted to the participant their elbow torque via the red circle (Fig 1). Additionally, a target blue circle was displayed corresponding to a torque value slightly larger than the estimated maximum that the participant could produce to provide further motivation.

### D. Detection Threshold

**Fig. 2.**
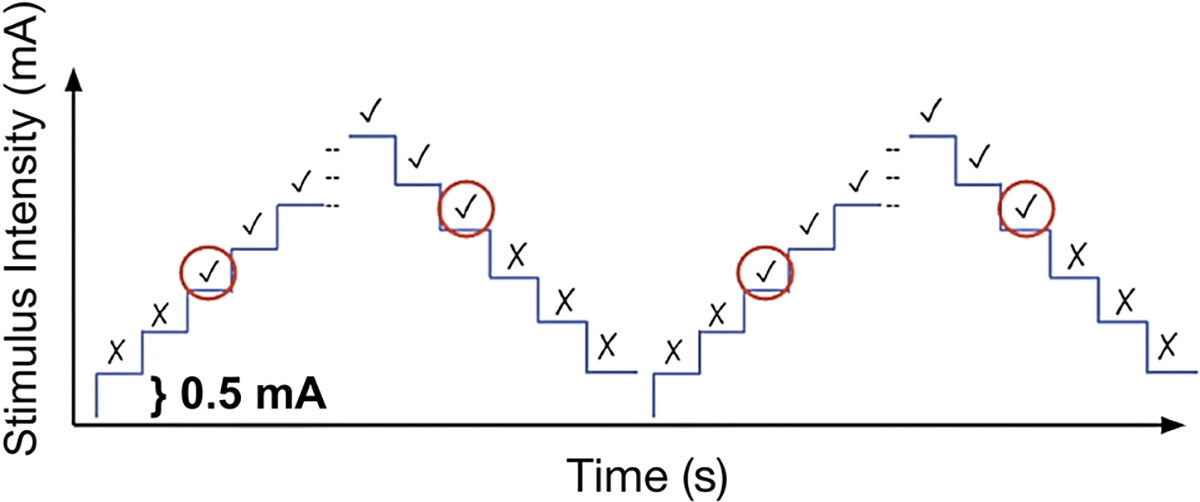
Schematic of the Staircase Method. A ’√‘ and ’×‘ indicates when the participant could and could not detect the electrotactile stimulus, respectively. The circled response indicates the first intensity at which the participant could or could not detect the electrotactile stimulus three times consecutively. The detection threshold was defined as the mean of the four circled values. Each step indicates an increase or decrease of 0.5 mA.

The participant’s detection threshold was recorded using a staircase method (Fig 2). The custom software automatically delivered the electrotactile stimulus at randomized time intervals to prevent the participant from predicting when the stimulus was given. The random time intervals were sampled from a uniform distribution between 2 and 5 seconds. The participant indicated verbally with a “yes” when the electrotactile stimulus was detected. When a stimulus was given and not detected, the participant did not respond and this failure to detect the stimulus was logged by the experimenter. The electrotactile stimulus strength was manually elevated by 0.5 mA intervals during the upward staircase until the participant indicated that the electrotactile stimulus was detected three consecutive times. The stimulation magnitude then increased by 1 mA from the last stimulus detected before beginning the downward staircase (i.e. if the last stimulus detected was 5 mA, the downward staircase began at 6 mA). The stimulus magnitude was then decreased by 0.5 mA intervals until the participant did not detect the stimulus three times in a row (i.e. no response). The value extracted from the upward staircase was the stimulus magnitude of the first time it was detected by the participant. The value obtained from the downward staircase was the stimulus magnitude of the last stimulus that was detected by the participant before no stimulus was detected three times in a row. This upward/downward staircase process was repeated one more time to obtain four values. These values were then averaged to identify the participant’s detection threshold.

For the elbow flexion conditions, the electrotactile stimulation only occurred when the participant generated the torque within the desired range. If the elbow flexion torque surpassed the desired range, audio feedback prompted the participant to relax, indicated that the torque generated was out-of-bounds, and the electrotactile stimulation stopped. Subsequently, the audio feedback informed the participant to flex again.

### E. Experimental Procedure

The participant was asked to avoid exercising their arms 24 hrs prior to the study to avoid muscle fatigue. The experimental session spanned approximately 4 hrs. After consenting to partake in the study, the participant was situated in the experimental setup. The participant’s MVT was first quantified. Next, a suprathreshold stimulus was delivered to the testing locations to ensure the participant knew how the stimulus should feel. Following, the detection threshold was identified at the fingertip and elbow of the testing arm. The detection threshold of the electrotactile stimulus was measured for three motor activation conditions: 1) at rest (baseline) and 2) isometrically flexing at the elbow to 25% of the participant’s MVT and 3) 50% of the participant’s MVT. To evaluate the residual effect of the motor activation on the detection threshold, the participant performed the baseline detection task before (pre-baseline) and after (post-baseline) the 25% and 50% MVT elbow flexion conditions. This testing procedure was performed for both arms. Presentation order of the arm first tested, stimulation location, and elbow flexion condition was randomized across participants using a Latin square randomization scheme.

### F. Torque Generation

To quantify participant variability in maintaining the elbow flexion torque, we used the coefficient of variation (CV). For each participant, we calculated the mean and standard deviation of the torque generated about the elbow when the desired range of the elbow flexion torque was reached. The CV of the torque generated was calculated as the percentage ratio between the standard deviation and mean.

### G. Statistical Analysis

Our primary goal was to determine whether detection of the electrotactile stimulus depended on the interactive effect of the arm and extent of motor activation. We first compared motor generation between the arms to address potential differences that may be relevant to the detection. The strength (MVT) in elbow flexion between arms was compared using a pairwise t-test. To address whether the variability in maintaining the flexion torque significantly differed depending on the arm and level of motor activation, we fit the CV results to a linear mixed-effects model. To address our primary goal of understanding the interactive effect of arm and motor activation on detection of the electrotactile stimulus, we fit the detection threshold outcome to a linear mixed-effects model as well.

For both linear mixed-effects models, motor activation condition, testing arm, and their interaction were fixed effects and participant was a random effect. An analysis of variance (ANOVA) was performed on both models using a hierarchical approach in which non-significant interaction terms were removed followed by non-significant main effect terms. Significantly differing levels were determined for significant main effects using post-hoc pairwise comparisons with a Holm correction to account for the multiple comparisons.

## III. RESULTS

Below we summarize our results describing the participants’ ability to maintain a steady torque and simultaneously detect an electrotactile stimulus. These findings are presented for each arm and motor activation condition.

### A. Torque Generation

Participant strength (MVT) in elbow flexion for the dominant and non-dominant arms was 59.5±14.1 Nm and 60.6±16.9 Nm, respectively. The MVT in elbow flexion did not differ significantly between arms (t(7)=0.680, p=0.518).

The average elbow torque recorded in the dominant arm at pre- and post-baseline was −0.1±0.1 Nm and 0.0±0.2 Nm, respectively. The average elbow torque recorded in the non-dominant arm at pre- and post-baseline was 0.0±0.2 Nm and 0.0±0.3 Nm, respectively. During the 25% MVT condition, participants generated about the dominant and non-dominant elbows 14.5±3. Nm and 14.9±4.2 Nm, respectively. During the 50% MVT condition, participants generated 27.8±6.2 Nm and 28.5±8.2 Nm about the dominant and non-dominant elbows, respectively.

**Fig. 3.**
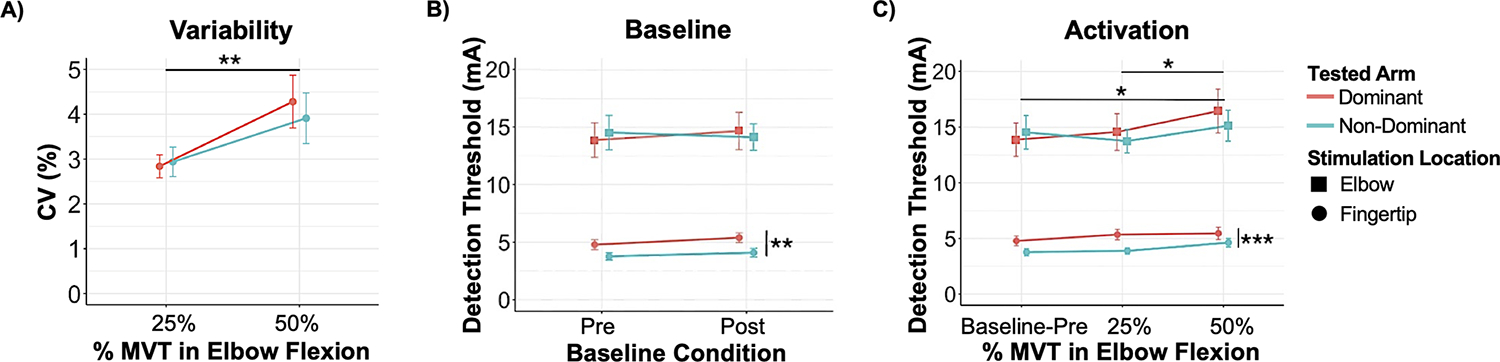
Elbow Flexion Variability and Detection Threshold **A.** Mean and standard error of participants’ CV when flexing about the elbow to 25% and 50% MVT in elbow flexion. **B.** Mean and standard error of participants’ detection threshold at the fingertip (circle) and elbow (square) pre- and post- baseline. **C.** Mean and standard error of the participants’ detection threshold during pre-baseline and the elbow flexion conditions at the fingertip (circle) and at the elbow (square). Data for the dominant and non-dominant arms are identified by red and blue circles, respectively. (*: p<0.050, **: p< 0.010, ***: p<0.001).

Next, we summarize the participants’ stability in generating the torques (Fig 3A). For the 25% MVT condition, the CV was 2.8±0.7% and 2.9±0.9% in the dominant and non-dominant arms, respectively. For the 50% MVT condition, the CV was 4.3±1.7% and 3.9±1.6% in the dominant and non-dominant arms, respectively. There was not a significant interaction of arm and motor activation condition on the CV (F(1, 21)=0.44, p=0.514). Additionally, the CV did not significantly differ between the arms (F(1, 22=0.15), p=0.703). However, the CV was greater at 50% than 25% MVT (F(1,23)=12.44, p=0.002). These results indicate that the level of motor activation affected the variability of the elbow torque generated, whereas a significant impact of arm dominance was not found.

### B. Detection Threshold At Rest

Fig 3B summarizes the detection threshold among participants at each arm for both baseline conditions.

For the fingertip, the detection threshold in the dominant arm was 4.8±1.2 mA and 5.4±1.2 mA in the pre- and post-baseline conditions. The detection threshold at the fingertip in the non-dominant arm was 3.8±0.9 mA and 4.1±1.1 mA in the pre- and post-baseline conditions. The detection threshold for the fingertip did not significantly differ at baseline before and after the motor activation conditions (F(1,22)=2.15, p=0.157), but differed significantly between arms (F(1,23)=12.45, p=0.002).

For the elbow, the detection threshold in the dominant arm was 13.9±4.2 mA and 14.7±4.6 mA in the pre- and post-baseline conditions. The detection threshold at the elbow in the non-dominant arm was 14.6±4.3 mA and 14.1±3.3 mA in the pre- and post-baseline conditions. The detection threshold at the elbow was not significantly affected by the arm (F(1,22)=0.02, p=0.882) or when the baseline condition was assessed (F(1,22)=0.23, p=0.639).

Combined, these results did not find differences in the ability to detect the electrotactile stimulus when comparing results before and after the motor activation testing.

### C. Detection Threshold with Elbow Flexion

Fig 3C summarizes the detection threshold among participants at each arm for every motor activation condition.

At the fingertip, relative to the detection threshold at baseline in the dominant arm (4.8±1.2 mA), the 25% and 50% MVT conditions increased by 0.5±1.8 mA and 0.7±1.9 mA, respectively. Relative to the detection threshold at baseline in the non-dominant arm (3.8±0.9 mA), the 25% and 50% MVT conditions increased by 0.1±1.2 mA and 0.8±1.4 mA respectively. There was not a significant interaction between the arm and elbow flexion torque (F(2,35)=0.50, p=0.611), indicating that activation-related tactile gating did not differ between the arms. The detection threshold at the fingertip did not significantly differ between the three activation conditions (F(2,37)=2.87, p=0.069). However, the detection threshold at the fingertip differed between the arms (F(1,37)=17.78, p<0.001), being greater at the dominant arm (5.19±1.3 mA) than the non-dominant arm (4.09±1.0 mA) across all motor activation conditions.

At the elbow, relative to the detection threshold at baseline in the dominant arm (13.9±4.2 mA), the 25% and 50% MVT conditions increased by 0.7±6.3 mA and 2.6±6.9 mA, respectively. Relative to the detection threshold at baseline in the non-dominant arm (14.5±4.3 mA), the 25% MVT condition decreased by −0.7±5.2 mA and the 50% MVT condition increased by 1.6±5.8 mA. There was not a significant interaction between the arm and elbow flexion torque (F(2,35)=1.98, p=0.154). Yet, motor activation did affect the detection threshold at the elbow (F(2,38)=6.09, p=0.005). Specifically, the detection threshold for the pre-baseline (14.2±4.1 mA) and 25% MVT (14.2±3.8 mA) conditions were smaller than the the 50% MVT (15.8±4.7 mA) condition. A significant difference was not obtained between the pre-baseline and 25% MVT (p=0.996) conditions. The arm did not significantly impact the detection threshold at the elbow (F(1,37)=1.29, p=0.264).

Combined, there was an impact of arm dominance on the detection threshold that is limited to the fingertip, and an effect of motor activation that is limited to the elbow.

## IV. DISCUSSION

Studies show that the left and right brain hemispheres specialize in various aspects of sensorimotor control processes, resulting in arm dominance [5], [22]. This laterality effect is well-documented in motor control literature. Specifically, the left hemisphere in right-arm dominant individuals is proposed to be specialized for predictable conditions, while the right hemisphere in this same population is specialized to stabilize during unpredictable events. In the context of sensorimotor control, our goal in this study was to determine whether the impact of brain laterality extended to the gating of tactile stimuli during motor actions. We observed gating of electrotactile stimuli at the elbow during the elbow flexion task, but not at the fingertip. Hence, our results do not reveal an impact of brain lateralization on how sensory signals are modulated in older adults during voluntary motor activation. Below we discuss the findings, limitations, and future directions.

### A. Motor Activation

Prior to considering sensorimotor perception, we needed to address the ability of our participants to generate and maintain the desired torques. Our results did not reveal differences in strength between the dominant and non-dominant arms. Additionally, when generating isometric flexion torques about the elbow, the variability in maintaining the torque, i.e. coefficient of variation in the torque generated, was similar between arms. In contrast, previous studies reported an effect of arm dominance on the stable control of postures [6], [26]. Our lack of a significant effect may be due to the simple nature of our task; the elbow flexion task was isometric, and individuals were given visual feedback and ample time to generate the desired torques. Given that our participants were older adults, the lack of differences in motor activation between the arms could also be due to an age-related reduction in hemispheric asymmetry [27]; the dominant arm becomes less superior in motor control than the non-dominant arm with aging [28]. Between the two elbow flexion conditions, the coefficient of variation was greater when individuals generated a greater magnitude of torque about the elbow. This result is in alignment with previous studies suggesting an increase in force variability with greater motor output [29], [30]. These findings demonstrate a significant impact of motor activation on torque control, whereas an impact of arm dominance was not apparent in our tested older adults.

### B. Detection of Electrotactile Stimuli

Our primary research goal was to determine whether motor activation impacted sensory gating differently depending on arm dominance. As a proxy for sensory gating, given our testing in humans, we used the outcome measure of detection threshold. We did not observe that the extent of sensory gating during motor activation differed depending on the arm dominance. Based on electrophysiological studies in animal models, activation-related gating of sensory signals can occur at subcortical and cortical sites along the somatosensory pathway [10], [31]. Arm dominance, in contrast, is thought to be a consequence of brain lateralization, which is a primarily cognitive process. As a result, this divergence in the neural origins of these two behaviors might explain the similar extent of sensory gating between the arms.

Even though we did not observe the interaction effect of arm dominance and motor activation on the detection threshold of the electrotactile stimuli, we did find that sensitivity to detect electrotactile stimuli was better at the middle fingertip of the non-dominant hand than the dominant hand. This difference in the detection threshold of electrotactile stimuli between arms was not observed at the elbow. Greater usage of the dominant hand than the non-dominant hand could have contributed to the observed difference [32]. For example, the fingertip of the middle finger in the dominant hand might have more calluses. In contrast, the skin around the elbow’s olecranon area is less likely to be subjected to such differences caused by usage. Additionally, the non-dominant arm has been shown to be more accurate in proprioception [33], [34] and perceiving the direction of tactile stimuli [35]. As a result, the difference in detection threshold between the hands observed in our study could be a reflection of the superiority of the non-dominant hand in somatosensory perception.

We also observed that the level of motor activation impacted the detection threshold of electrotactile stimuli at the elbow, but not at the fingertip. Similar results have been reported in previous psychophysical studies in humans, indicating that sensory signals located closer to the area of motor activation were gated to a greater extent [16], [18]. Post et al. [16] asked participants to perform an isometric elbow flexion task while detecting vibrotactile stimuli applied at the finger, thenar eminence, and forearm. An increase in the detection threshold was observed at all three locations, with the greatest increase at the forearm [16]. In our study, the area around the elbow is closer to the site of motor activation, i.e. elbow flexors, than the fingertip. As such, it was expected that the detection threshold at the elbow would increase to a greater extent than that at the fingertip during motor activation, as relative to the baseline. It is also worth noting that, given the placement of the electrodes in our work, it is the median nerve stimulated at the fingertip while it is the ulnar nerve stimulated at the elbow. It is unclear whether somatosensory signals are gated differently between sensory nerves and, consequently, contribute to our observed results.

The increase in the detection threshold at the elbow is likely a result of backward masking [18] and not prediction [15], [19], [36]. The stimulus we provided, i.e. electrotactile stimulus at the fingertip, was not relevant for the task, i.e. elbow flexion. Additionally, the electrotactile stimulus was not predictable as it was applied externally at random time intervals. As such, it is reasonable to assume that the stimulus was not included in the individual’s sensorimotor prediction and would not be modulated in a task-dependent manner.

We highlight that the participants included in this study are older adults. Processing of sensory stimuli in this population differs from that in young adults. For example, older adults have lower nerve conduction velocities, increased detection thresholds for touch and pressure, and greater suppression of tactile signals during reaching than young adults [37], [38], [39]. Our findings have implications when considering individuals with stroke or other neurological impairments who tend to be older; yet, it is unclear whether our findings can generalize to a younger population.

### C. Limitations

Our findings may be limited in their ability to detect a significant impact of motor activation at the fingertip due to the small sample size. That said, the effect size may be small at the fingertip given a significant effect was observed at the elbow. Our study findings also are limited to right-hand dominant participants. Future work can determine whether our findings extend to left-hand dominant participants. Finally, our detection thresholds may have been impacted by variables such as temperature and humidity, which have been shown to affect the flow of sensory signal transmission. Future work can use a more controlled experimental setup, including measuring and controlling limb temperature, to ensure that these variables are comparable at each arm.

### D. Concluding Remarks and Significance

The ability to detect externally-applied tactile stimuli during sensorimotor tasks is important for successfully performing activities of daily living. Our results indicate that older adults are more sensitive to an electrotactile stimulus applied at the fingertip of their non-dominant hand than their dominant hand and are less sensitive to an eletrotactile stimulus at the elbow during elbow flexion. Our finding regarding the impact of motor activation on tactile perception is important when considering the design of haptic interfaces. For example, when designing a virtual environment with haptic feedback, as the individual engages in motor activity, the potential saturation of one’s perception of tactile information can be considered. Additionally, as the electrotactile stimulation is a viable sensorimotor training approach for individuals with stroke [40], [41], our results on older adults may have clinical implications for better incorporating the use of electrotactile stimulation in rehabilitation pratice. Lastly, since object manipulation is done primarily with the hands, future work can employ a motor activation task that involves both hands and investigates whether changes in somatosensory perception occur when this more distal and localized site is activated.
